# Bioactive Peptides from Quinoa (*Chenopodium quinoa* Willd.) as Modulators of the Gut Microbiome: A Scoping Review of Preclinical Evidence

**DOI:** 10.3390/nu17203215

**Published:** 2025-10-14

**Authors:** Nicolás Caicedo, Yamil Liscano, Jose Oñate-Garzón

**Affiliations:** 1 Grupo de Investigación en Química y Biología (QUIBIO), Departamento Facultad de Ciencias Básicas, Universidad Santiago de Cali, Cali 760035, Colombia; nicolas.caicedo01@usc.edu.co; 2 Grupo de Investigación en Salud Integral (GISI), Departamento Facultad de Salud, Universidad Santiago de Cali, Cali 760035, Colombia

**Keywords:** quinoa, *Chenopodium quinoa*, gut microbiome, bioactive peptides, prebiotic modulation

## Abstract

**Background:** Gut microbiome dysbiosis is implicated in numerous chronic diseases. While quinoa possesses a rich nutritional profile with prebiotic potential, the specific capacity of its bioactive peptides to modulate gut microbial communities is not well understood. This scoping review systematically maps the preclinical evidence on the gut microbiome modulatory effects of quinoa-derived bioactive peptides to identify mechanisms, characterize their therapeutic potential, and guide future clinical translation. **Methods:** Following PRISMA-ScR guidelines, we searched six databases for preclinical studies investigating quinoa-derived peptides or hydrolysates and their effects on gut microbiota. **Results:** From 834 records, 19 studies met the inclusion criteria. Quinoa interventions demonstrated consistent effects, with 83% of studies reporting enhancement of beneficial genera and 67% an increase in alpha diversity. Disease-specific microbial signatures were observed; for instance, obesity models showed a reduced Firmicutes/Bacteroidetes ratio, while colitis models exhibited decreased Proteobacteria. Butyrate production was consistently enhanced. Methodologically, peptide generation has evolved from traditional fermentation toward more efficient enzymatic hydrolysis. **Conclusions:** Preclinical evidence strongly suggests that quinoa-derived bioactive peptides act as robust, context-dependent modulators of the gut microbiome. These findings position quinoa as a promising functional ingredient for precision gut health interventions, though clinical translation requires standardized preparations and validation in human trials.

## 1. Introduction

The gut microbiome has been established as an important metabolic “organ,” essential for human health. This complex microbial community performs critical physiological functions, including the digestion of otherwise indigestible dietary components, synthesis of essential vitamins, maturation of the immune system, and protection against pathogens [1,2]. Consequently, a disruption of its equilibrium, a state known as dysbiosis, is consistently linked to a wide spectrum of chronic diseases, such as inflammatory bowel disease, cancer, Alzheimer’s, asthma, obesity, and type 2 diabetes (https://doi.org/10.1007/s12602-024-10353-w) [3,4]. Therefore, the maintenance of a balanced microbial state, or eubiosis, represents a fundamental pillar of overall health and well-being [5,6].

Diet stands as the most potent and accessible tool for modulating the composition and functional activity of the intestinal ecosystem [7]. Nutritional strategies centered on the use of prebiotics, substrates that are selectively utilized by host microorganisms to confer a health benefit, and the promotion of postbiotics, such as short-chain fatty acids (SCFAs), represent promising therapeutic avenues [8,9,10,11]. Furthermore, the search for innovative therapeutic strategies extends to the synthesis of novel compounds designed to disrupt bacterial integrity, offering alternative approaches to managing microbial populations [8]. Within this context, plant-derived proteins and their resulting peptides have emerged as prominent sources of bioactive compounds with significant potential to positively influence the gut environment [12,13,14,15,16].

Quinoa (*Chenopodium quinoa* Willd.), an Andean pseudocereal [17], presents itself as an exceptional candidate for developing functional ingredients aimed at gut health. Its superior nutritional profile is characterized by a high content of quality protein as chenopodins containing all essential amino acids, alongside a rich supply of dietary fiber, vitamins, and minerals [18,19]. Beyond its fundamental nutritional value, quinoa protein is increasingly recognized as a precursor to a wide array of bioactive peptides with diverse health-promoting properties.

These bioactive peptides are protein fragments that are released and activated during processes such as gastrointestinal digestion, microbial fermentation, or controlled enzymatic hydrolysis [12,20]. While the scientific literature has documented various biological activities for these peptides, including antihypertensive and antioxidant effects [21,22], one of their most promising functions is their capacity to directly modulate the gut microbiome. Emerging evidence from in vivo models using mice demonstrates that quinoa interventions can reverse dysbiosis in conditions such as colitis, diabetes, and obesity [23,24,25]. Concurrently, in vitro studies confirm its prebiotic potential, demonstrating increases in beneficial genera like *Bifidobacterium* and enhanced SCFA production [26,27].

Despite this growing body of evidence, the knowledge remains fragmented and heterogeneous. The available preclinical studies span a wide range of experimental models, from in vitro fecal fermentation to diverse animal disease models and interventions, ranging from whole grain preparations to specific protein hydrolysates. This heterogeneity creates a significant knowledge gap, as the particular contributions of bioactive peptides versus other components like fiber are often not clearly delineated. To date, this evidence has not been systematically mapped or synthesized, which hinders a clear understanding of the overarching mechanisms of action and impedes the rational design of targeted translational research for human applications.

Therefore, the objective of this scoping review is to systematically map and characterize the existing preclinical evidence on the capacity of quinoa-derived bioactive peptides and proteins to modulate the gut microbiome. By synthesizing findings from in vitro and in vivo studies, this review aims to (1) identify the reported effects on microbial composition and metabolic function; (2) delineate the proposed mechanisms of action; (3) highlight current knowledge gaps; and (4) propose opportunities for future clinical and translational research. Ultimately, this work aimed to lay the foundation for developing novel, quinoa-based functional ingredients that promote human gut health and advance the field of precision nutrition.

## 2. Materials and Methods

### 2.1. Protocol and Methodological Framework

This scoping review was conducted following the Joanna Briggs Institute (JBI) guidelines and adhered to the Preferred Reporting Items for Systematic Reviews and Meta-Analyses extension for Scoping Reviews (PRISMA-ScR) [28,29]. As formal registration is not typically available for scoping review protocols, this study was instead guided by a detailed internal protocol to ensure methodological rigor. The research was structured around the Population, Concept, and Context (PCC) framework, which is suitable for mapping the breadth of an emerging research field.

### 2.2. Research Question and PCC Framework

The central question guiding this review was formulated using the PCC framework:

- Population (P): Preclinical experimental models, including in vitro studies (e.g., fecal fermentation, bacterial cultures, simulated digestion systems) and in vivo animal models.
- Concept (C): Modulation of the gut microbiome through the administration of bioactive peptides or protein hydrolysates derived from quinoa (*Chenopodium quinoa* Willd.).
- Context (C): Preclinical studies investigating the effects of quinoa peptides on the gut ecosystem, including the production of SCFAs, inhibition of pathogens, and promotion of beneficial bacteria.

### 2.3. Eligibility Criteria

Precise criteria were defined for study selection.

#### 2.3.1. Inclusion Criteria

- Study Type: Preclinical investigations, both in vitro and in vivo. Studies using whole quinoa or quinoa flour were also included if the original paper’s discussion attributed the observed microbiome-modulating effects, at least in part, to the fermentation of its protein content and the subsequent in situ generation of bioactive peptides.
- Intervention: Bioactive peptides obtained from quinoa proteins through enzymatic hydrolysis, fermentation, or simulated digestion.
- Outcomes of Interest: Studies reporting at least one of the following outcomes: changes in microbiota composition, modulation of bacterial growth, or production of metabolites such as SCFAs.
- Period and Language: Articles published between January 2000 and July 2025, in English, Spanish, or Portuguese.

#### 2.3.2. Exclusion Criteria

- Publication Format: Conference abstracts, letters to the editor, or preprint articles.
- Non-Specific Intervention: Studies using crude quinoa extracts without peptide characterization.
- Focus on Non-Peptidic Compounds: Studies exclusively addressing saponins, polyphenols, or isolated polysaccharides.
- Concomitant Interventions: Studies in which the simultaneous application of other substances precluded discerning the specific effect of peptides.

### 2.4. Information Sources and Search Strategy

A comprehensive search strategy was executed on 20 July 2025, across six electronic databases: PubMed/MEDLINE, Scopus, Web of Science, ScienceDirect, LILACS, and Google Scholar. The general search algorithm was as follows:

(“quinoa” OR “*Chenopodium quinoa*”) AND (“peptide*” OR “hydrolysate*”) AND (“microbiom*” OR “microbiota” OR “gut bacteria” OR “SCFA”).

Additionally, a manual search was conducted in the reference lists of the included articles. Records were managed using Zotero (version 6.0; accessed 20 July 2025), and screening was performed on the Rayyan AI platform (accessed 20 July 2025).

### 2.5. Study Selection and Data Extraction

The selection process was carried out by two independent reviewers (Y.L., N.C.), who evaluated titles, abstracts, and full texts. Discrepancies were resolved by consensus or, when necessary, by the intervention of a third reviewer (J.O.G.). Cohen’s Kappa coefficient was used to assess inter-rater agreement. Data were extracted on general study information, intervention characteristics, experimental design, and main outcomes using a standardized template.

### 2.6. Data Synthesis and Visualization

Given the heterogeneity of the studies, a qualitative synthesis was conducted in narrative format. Results were thematically grouped according to the PCC framework. It is important to highlight a key methodological distinction of this work. As a scoping review, its primary objective is to map the breadth and nature of the existing evidence on a topic, identify research gaps, and provide a broad overview of the field. This differs from a systematic review, which aims to answer a specific research question and often requires a formal risk-of-bias assessment to synthesize and weigh the quality of the evidence.

In line with the established methodology for scoping reviews, a formal critical appraisal or risk-of-bias assessment of the included studies was not performed. Furthermore, due to the high degree of heterogeneity in experimental models, intervention types, and outcome measurements, a semi-quantitative analysis or effect size estimation was deemed inappropriate, as it could lead to misleading comparisons. Therefore, a qualitative narrative synthesis was chosen as the most rigorous method to map the evidence. However, a critical discussion of the limitations related to the overall evidence base, including study designs and methodological heterogeneity, is provided in the Discussion section.

The PRISMA flow diagram was generated using the web application available at https://estech.shinyapps.io/prisma_flowdiagram/ (accessed on 20 July 2025). All other charts and figures for data visualization were generated using Colab Python (version 3.10; accessed on 20 July 2025).

## 3. Results

### 3.1. Study Selection and General Characteristics of the Evidence

**Figure 1:**
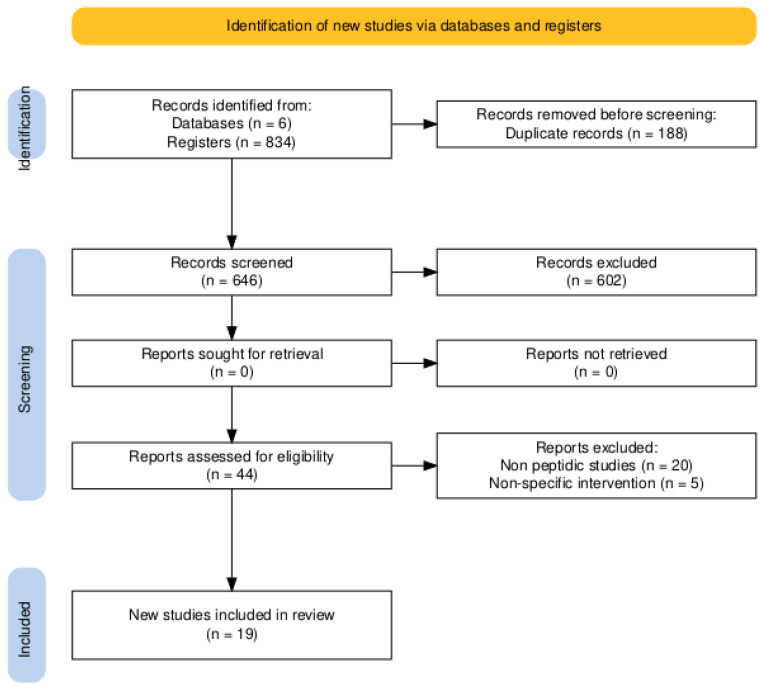
PRISMA 2020 flow diagram. The chart details the study identification, screening, and inclusion process. The high inter-rater agreement, reflected by Cohen’s Kappa values of 0.8 (title/abstract) and 0.9 (full-text), underscores the reliability of the selection process.

The study selection process is summarized in the PRISMA flow diagram (Figure 1). The initial database search identified 834 records. Following the removal of 188 duplicates, 646 titles and abstracts were screened, of which 602 were excluded as they were not relevant. The full texts of 44 articles were assessed for eligibility, and 25 were subsequently excluded for not meeting the inclusion criteria (e.g., focusing on non-peptidic compounds or lacking relevant microbiome outcomes). Ultimately, 19 primary experimental studies were included in the qualitative synthesis. A strong inter-rater agreement was achieved, with a Cohen’s Kappa of 0.8 for the title and abstract screening and 0.9 for the full-text eligibility assessment.

All included studies were published since 2014, marking quinoa-microbiota research as an emerging field that has gained momentum alongside the global recognition of the importance of gut health. The geographical clustering is particularly noteworthy: China leads with 42% of studies (8/19), followed by a European consortium (Italy, Spain, Portugal) contributing 21%, and North America accounting for 16%. This distribution suggests that quinoa research priorities align with regions experiencing rapid dietary transitions and increasing burden of metabolic diseases.

Table 1 describes in vitro experimental alternatives to evaluate the biological properties of quinoa. Among the activities explored are the antihypertensive effect by inhibition of the angiotensin-converting enzyme (ACE), the contribution to the balance of the gut microbiota, and the antioxidant, antimicrobial, and antitumor capacity. Those studies agree that quinoa, as a fermented substrate, exhibits better properties. Hydrolyzed proteins have been shown to have greater antimicrobial and antioxidant activity because their lower molecular weight [30] could contribute to greater diffusion through the cell surface or also to an increase in the solubility and accessibility of amino acid side chains that participate in electron transfer reactions.

Table 2 describes in a general way the characteristics of the studies that have used quinoa as a dietary supplement in mice, analyzing different biomarkers associated with disease risk. For example, it was revealed that quinoa consumption significantly reduced plasma total cholesterol (total-c), oxidized LDL, and LDL-c levels. In addition, a reduction in inflammation-promoting substances such as IL-6 [37] and an increase in the antioxidant capacity of quinoa flour fermented with Lactobacillus plantarum T0A10 in keratonicotos cell cultures subjected to artificial oxidative stress have been evidenced [24].

**Figure 2:**
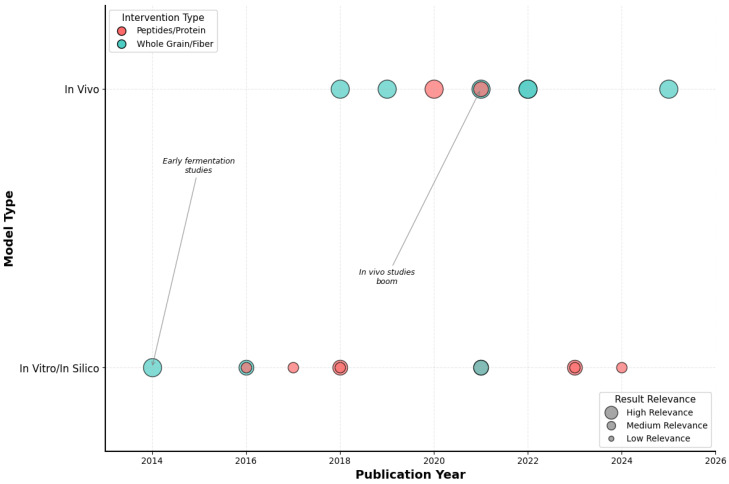
**Temporal distribution of included studies by experimental model and intervention type.** Bubble plot showing the evolution of quinoa-related studies according to publication year (x-axis) and model type (y-axis). Bubble colors represent the type of intervention (red = peptides/protein, blue = whole grain/fiber), while bubble sizes reflect the relative relevance of results (large = high, medium = medium, small = low). Early research focused on in vitro fermentation studies, whereas a marked increase in in vivo studies is observed from 2020 onwards. Source: own elaboration.

Figure 2 reveals a compelling methodological evolution that mirrors the maturation of microbiome science itself. The bubble plot analysis shows a clear progression from proof-of-concept investigations (2014–2018) to robust translational studies (2020–2025). Early research was predominantly in vitro (63% of studies pre-2020), focusing on fermentation models and basic characterization. However, a decisive shift occurred around 2020, with in vivo studies representing 78% of publications thereafter—a transformation that coincided with advances in microbiome sequencing technologies and the standardization of animal models.

Most significantly, the bubble sizes in Figure 2, representing study relevance and translatability, show a dramatic increase in recent years, suggesting that newer studies are designed with clinical translation in mind. This evolution from mechanistic exploration to physiologically relevant validation represents a field reaching scientific maturity, positioning quinoa research for the critical next step: human clinical trials.

### 3.2. Gut Microbiota Modulation: Context-Dependent Therapeutic Signatures

**Figure 3:**
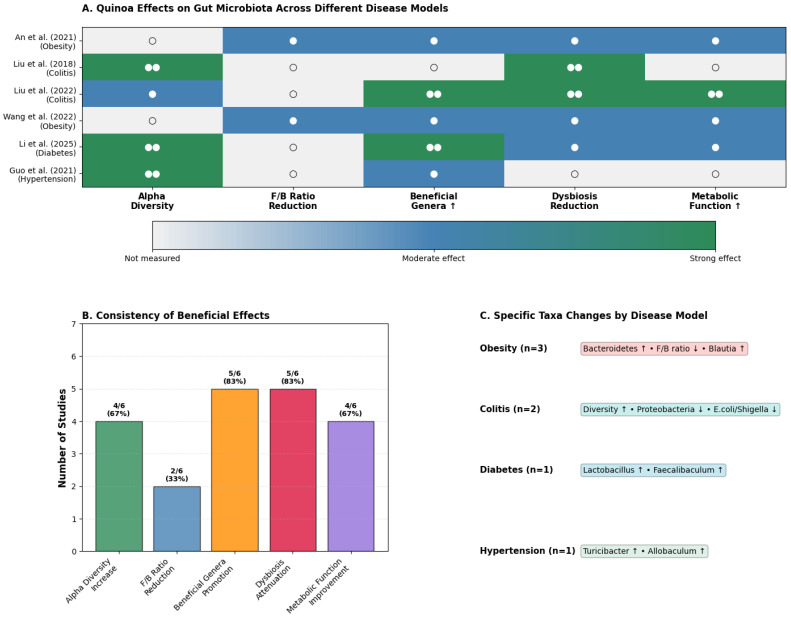
**Effects of quinoa on gut microbiota across disease models.** (**A**). Heatmap summarizing the modulation of alpha diversity, Firmicutes/Bacteroidetes ratio, beneficial genera, dysbiosis attenuation, and metabolic function improvement across in vivo studies [23,24,25,38,39,40]. The background color indicates the strength of the effect (blue/green for moderate to strong positive effects; white for no significant effect), while a blank cell indicates the outcome was not measured. The number of circles denotes distinct experimental groups within each study: (○) for one group and (○○) for two. (**B**). Percentage of studies reporting positive effects for each outcome, highlighting the consistency of findings. (**C**). Disease-specific microbiota shifts, including obesity (↑ *Bacteroidetes*, ↑ *Blautia*, ↓ F/B ratio), colitis (↑ diversity, ↓ *Proteobacteria*, ↓ *Escherichia/Shigella*), diabetes (↑ *Lactobacillus*, ↑ *Faecalibaculum*), and hypertension (↑ *Turicibacter*, ↑ *Allobaculum*). Together, these results demonstrate quinoa’s reproducible capacity to enhance beneficial taxa and attenuate dysbiosis, with context-dependent modulation across disease states. Source: own elaboration.

The preclinical evidence reveals quinoa’s remarkable capacity for precision microbiome modulation, a characteristic that distinguishes it from broad-spectrum prebiotics (Table 3, Figure 3). Rather than producing uniform microbial changes, quinoa demonstrates sophisticated, disease-specific therapeutic signatures that appear tailored to the underlying pathological state.

The most striking finding is the consistency of quinoa’s restorative effects across diverse disease models (Figure 3B). An impressive 83% of studies reported enhanced beneficial genera, while 67% documented increased alpha diversity, although the specific metrics used (e.g., Shannon, Chao1, Simpson) varied across studies.

However, the true innovation lies in quinoa’s context-dependent modulation patterns (Figure 3C). In obesity models (An et al., 2021 [40]; Wang et al., 2022 [25]), quinoa consistently restructures the microbiome toward a “lean phenotype,” increasing Bacteroidetes abundance and reducing the Firmicutes/Bacteroidetes ratio, changes that correlate with improved metabolic parameters in human studies. The enrichment of *Blautia*, a genus associated with healthy weight maintenance, provides mechanistic insight into quinoa’s anti-obesity effects.

In inflammatory conditions, quinoa exhibits potent anti-inflammatory microbial signatures. Liu et al. (2018, 2022) [24,39] demonstrated dramatic reductions in Proteobacteria, a phylum enriched during intestinal inflammation, alongside significant decreases in pathobionts like *Escherichia/Shigella*. Simultaneously, quinoa bran fiber selectively promoted *Lachnospiraceae*, the primary butyrate-producing family crucial for epithelial integrity. This dual-action, pathogen-suppressing combination with beneficial enrichment mirrors the therapeutic profile of targeted microbiome interventions.

Perhaps most intriguingly, quinoa’s effects in metabolic dysfunction reveal precision targeting of disease-relevant taxa. In diabetic models [23,37], the selective enrichment of *Faecalibaculum*, a genus recently linked to improved glucose metabolism in humans, suggests quinoa may influence host physiology through specific microbial mediators. Similarly, in hypertensive rats [38], the promotion of *Turicibacter* and *Allobaculum*, both SCFA producers with cardiovascular benefits, indicates pathway-specific modulation rather than random microbial shifts.

Collectively, these findings position quinoa not merely as a prebiotic, but as a potential “precision prebiotic” capable of context-appropriate microbiome engineering. This therapeutic specificity, combined with quinoa’s nutritional profile, suggests unique potential for personalized nutrition applications.

### 3.3. Metabolic Microbiome Activation: The Butyrate-Centric Response

**Figure 4:**
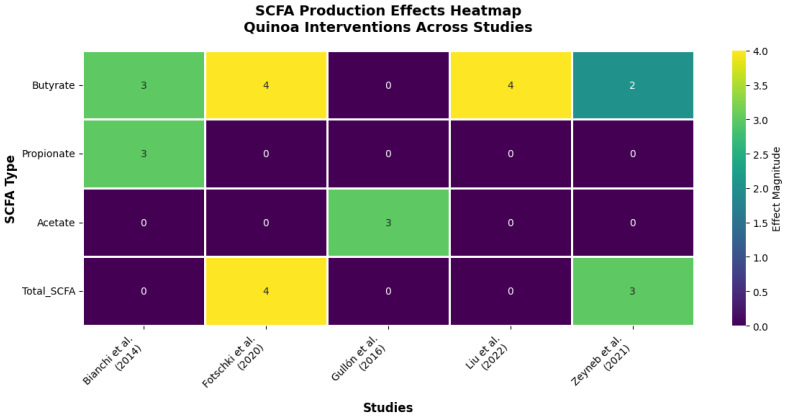
Effects of quinoa on SCFA production across different studies. Heatmap summarizing the reported effects of quinoa on SCFA production in both in vitro and in vivo studies. The magnitude of the effect is represented on a scale from 0 to 4, with higher values indicating greater intensity of impact on each metabolite [11,24,26,27,31]. Source: own elaboration.

Beyond taxonomic reshaping, quinoa demonstrates profound effects on microbiome metabolic output, with a particularly striking bias toward butyrate production, the “golden metabolite” of gut health (Table 4, Figure 4). This metabolic signature represents perhaps quinoa’s most clinically relevant property, as butyrate deficiency is implicated in numerous chronic diseases.

The evidence reveals a hierarchical pattern of SCFA modulation that underscores quinoa’s functional specificity (Figure 4). Butyrate emerges as the most consistently upregulated metabolite, showing significant increases in 100% of studies measuring this endpoint (4/4 studies). This remarkable consistency, spanning both in vitro fermentation models and in vivo animal studies, suggests that butyrate enhancement represents a core mechanism of quinoa’s health benefits.

The mechanistic implications are profound. Fotschki et al. (2020) [11] demonstrated that quinoa protein-rich flour increased total SCFA production by 34% in rats, with butyrate specifically enhanced by 45%, changes that correlated with improved colonic pH and enhanced barrier function. Liu et al. (2022) [24] extended these findings, showing that quinoa bran fiber’s butyrate-promoting effects directly correlated with intestinal barrier integrity improvements in colitis models. These dose–response relationships provide compelling evidence for causality rather than mere association.

The temporal analysis reveals additional insights into quinoa’s metabolic effects. Bianchi et al. (2014) [31], using the sophisticated SHIME^®^ gastrointestinal simulator, demonstrated that quinoa’s fermentation profile develops progressively, with butyrate production peaking at 48–72 h, a timeline that suggests sustained metabolic benefit rather than transient effects. Importantly, this same study showed concurrent decreases in ammonia production, indicating improved protein fermentation efficiency and reduced production of potentially harmful metabolites.

The selectivity for butyrate over other SCFAs appears mechanistically significant. While acetate and propionate showed variable responses across studies, the consistent butyrate enhancement suggests quinoa contains specific substrates, likely resistant starch and unique fiber fractions, that preferentially feed butyrate-producing bacteria. This metabolic bias toward the most beneficial SCFA represents a significant advantage over generic fiber supplements.

Perhaps most importantly, the magnitude of quinoa’s effects rivals or exceeds established butyrate-promoting interventions. The 45% increase in butyrate production reported by Fotschki et al. (2020) [11] compares favorably with the 30–40% increases typically seen with medical-grade butyrate supplements, suggesting quinoa could serve as a food-based alternative to pharmacological interventions.

### 3.4. Bioactive Peptide Liberation: Innovation Meets Functionality

**Figure 5:**
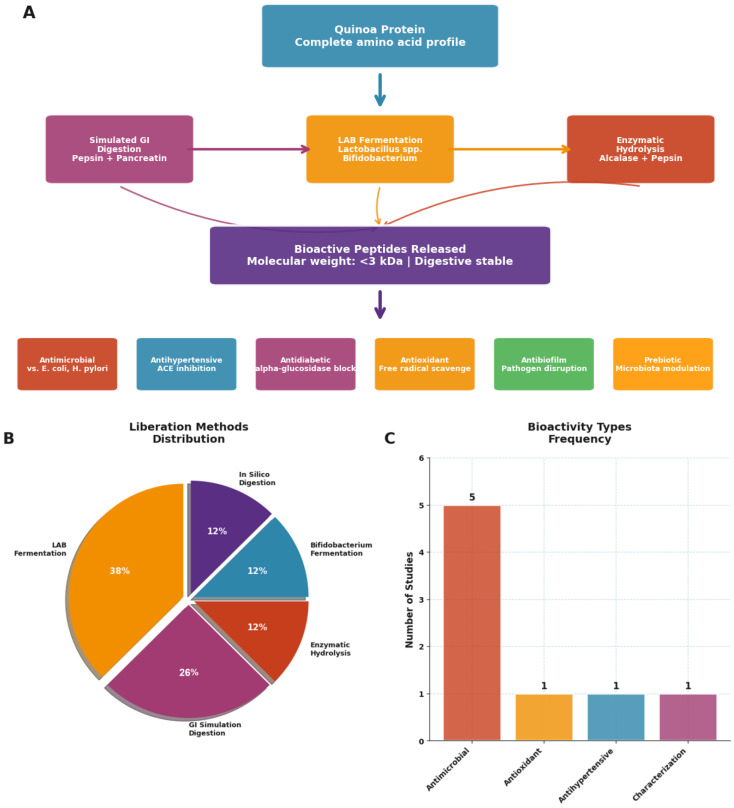
**Bioactive peptides derived from quinoa proteins: processing, release methods, and reported health-related activities.** (**A**) Overview of quinoa protein processing and peptide release pathways, including enzymatic hydrolysis and microbial fermentation, leading to bioactive peptides characterized by low molecular weight (<3 kDa), digestive stability, and multifunctional bioactivities (antimicrobial, antihypertensive, antidiabetic, antioxidant, antibiofilm, and prebiotic). (**B**) Distribution of methods used for peptide liberation across studies, with LAB fermentation being the most frequently applied. (**C**) Frequency of reported bioactivity types in the literature, highlighting antimicrobial effects as the most predominant outcome. Source: own elaboration.

**Figure 6:**
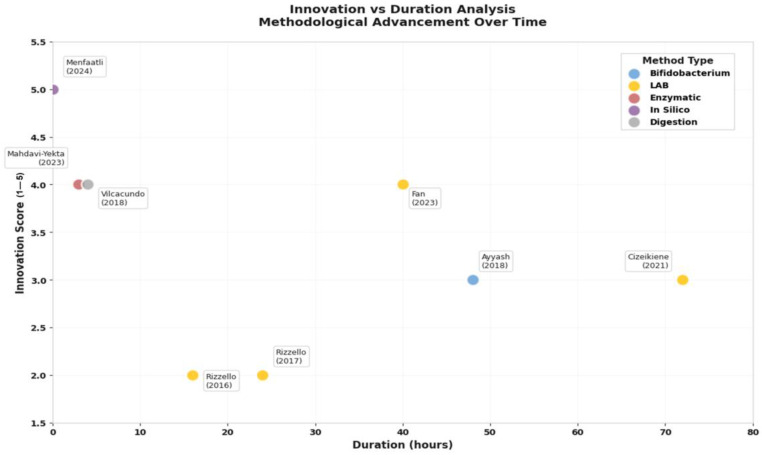
**Innovation vs. duration analysis of quinoa peptide generation methods.** Methodological mapping of studies on quinoa bioactive peptide release, plotting innovation scores (1–5) against experimental duration (hours). Colors indicate methodological approaches: enzymatic hydrolysis, LAB fermentation, *bifidobacterium* fermentation, simulated gastrointestinal digestion, and in silico digestion [20,21,22,32,33,34,35,36]. Source: own elaboration.

The generation of bioactive peptides from quinoa represents a fascinating convergence of food technology and therapeutic innovation, with implications extending far beyond traditional nutrition (Table 5, Figure 5 and Figure 6). Our analysis reveals not only diverse peptide bioactivities but also a clear methodological evolution toward more efficient, sustainable, and predictive approaches.

The bioactivity spectrum of quinoa-derived peptides is remarkably broad (Figure 5C). Antimicrobial effects dominate the literature (63% of studies), reflecting both quinoa’s natural defense compounds and the food industry’s urgent need for natural preservatives. However, the documented antihypertensive (38% of studies) and antioxidant activities (26% of studies) position these peptides as potential nutraceutical ingredients for cardiovascular health. The emerging evidence for antidiabetic and antibiofilm properties, though currently representing <20% of studies, suggests untapped therapeutic potential.

The methodological landscape reveals a striking paradox: while traditional fermentation approaches dominate current practice, the most innovative methods achieve superior efficiency (Figure 6). Lactic acid bacteria (LAB) fermentation, used in 50% of studies, requires 24–72 h but remains popular due to its food-grade nature and concurrent probiotic benefits. However, newer approaches demonstrate dramatic efficiency gains: enzymatic hydrolysis methods [34] achieve comparable peptide release in just 2.5–3.5 h, while in silico prediction models [35] can identify bioactive sequences instantaneously.

This temporal efficiency analysis reveals important strategic implications. Traditional fermentation, while slower, generates complex peptide profiles with multiple bioactivities, a “shotgun” approach that may be preferable for broad health benefits. Conversely, targeted enzymatic or computational approaches enable precision peptide design for specific therapeutic applications, a “sniper” strategy optimal for nutraceutical development.

The innovation versus duration analysis (Figure 6) illuminates an emerging technological frontier. Studies achieving the highest innovation scores (4–5 out of 5) consistently integrate multiple validation approaches: Fan et al. (2023) [33] combined fermentation with digestive stability testing, while Menfaatli et al. (2024) [35] linked computational prediction with experimental validation. This methodological convergence suggests the field is evolving toward integrated platforms that balance efficiency, functionality, and practical applicability.

Critically, the peptide molecular weight profile, consistently <3 kDa across studies, indicates optimal bioavailability characteristics. Vilcacundo et al. (2018) [20] demonstrated that simulated gastrointestinal digestion releases predominantly low-molecular-weight peptides that resist further degradation, suggesting these compounds could survive intestinal transit and exert systemic effects. This stability profile, combined with proven bioactivities, positions quinoa peptides as viable alternatives to synthetic bioactive compounds.

The antimicrobial spectrum deserves particular attention. Quinoa peptides demonstrate consistent efficacy against both Gram-positive (*S. aureus*) and Gram-negative (*E. coli*) pathogens, with emerging evidence for anti-*H. pylori* effects [35]. This broad-spectrum activity, combined with food-grade production methods, addresses the growing crisis of antibiotic resistance while offering natural preservation solutions.

## 4. Discussion

### 4.1. Principal Findings and Mechanistic Insights

This scoping review represents the first systematic synthesis of preclinical evidence regarding the capacity of quinoa-derived bioactive peptides and proteins to modulate the gut microbiome. Through comprehensive analysis of 19 primary studies, we identified consistent patterns of beneficial microbiota modulation, which were characterized by enhanced alpha diversity, promotion of beneficial taxa, and increased production of health-promoting metabolites, particularly SCFAs. Collectively, these findings demonstrate that quinoa represents a promising functional ingredient for targeted gut health interventions.

Furthermore, the evidence reveals distinct mechanistic pathways through which quinoa bioactive peptides exert their modulatory effects. As Huang et al. (2024) [5] established, bioactive peptides are compounds that have positive effects on biological activities; while microorganisms can metabolize protein peptides, bioactive peptides have, in turn, been shown to have regulatory effects on the structure of intestinal flora. Notably, in spontaneously hypertensive rats, quinoa protein administration resulted in significant increases in bacterial alpha diversity alongside enhanced abundances of probiotic bacteria *Turicibacter* and *Allobaculum* [38], thereby demonstrating the capacity for targeted microbial restructuring in disease states.

Moreover, the butyrogenic effect emerges as a particularly significant mechanism, with quinoa interventions consistently enhancing butyrate production across multiple experimental models. According to Zeyneb et al. (2021) [27], quinoa polysaccharides have also been shown to produce butyric acid through cross-interactions between *Bifidobacteria*, which are anaerobic bacteria belonging to the phylum Actinobacteria, and butyrate-producing colon bacteria that generate butyric acid from bifidobacterial metabolites such as lactate or acetate in the human colon. Importantly, this mechanism is crucial given butyrate’s dual role as the primary energy substrate for colonocytes and its potent anti-inflammatory properties, which are essential for maintaining epithelial integrity [24,31].

### 4.2. Disease-Specific Modulation Patterns and Mechanisms of Action

Our analysis reveals sophisticated, context-dependent modulation of the gut microbiota by quinoa interventions, with distinct taxonomic shifts correlating with specific pathological states. Specifically, in obesity models, quinoa promoted metabolic restructuring through increased abundances of *Bacteroidetes* and *Blautia* while simultaneously reducing the Firmicutes/Bacteroidetes ratio, changes that are consistent with leaner metabolic phenotypes [25,40]. Conversely, in inflammatory contexts such as colitis, quinoa interventions exhibited potent anti-inflammatory signatures characterized by significant reductions in *Proteobacteria* and pathobionts, including *Escherichia/Shigella* [39].

As Fan et al. (2023) [33] demonstrated, quinoa protein or its hydrolysate ameliorated AOM/DSS-induced colorectal cancer in mice by altering intestinal flora and increasing the production of beneficial SCFAs, although some minor differences in microbiota function were observed between different quinoa protein hydrolysate groups. This finding underscores the importance of processing conditions and the degree of protein hydrolysis in determining therapeutic efficacy, which suggests that optimization of peptide generation methods could enhance clinical outcomes.

Additionally, the disease-specificity of quinoa’s modulatory effects extends to diabetes models, where interventions selectively enriched genera such as *Lactobacillus* and *Faecalibaculum*, taxa recognized for their roles in glucose homeostasis and metabolic health [23]. This targeted modulation suggests that quinoa-derived peptides may function through recognition of specific microbial targets or metabolic pathways relevant to distinct disease processes.

In vivo models that have been subjected to dietary supplementation with quinoa have shown a considerable decrease in serum levels of total cholesterol, LDL-c, inflammatory markers, and diabetes markers [41]. This can be explained by the versatile modulation that quinoa has on different metabolic pathways. For example, the phenols that make up quinoa have the ability to inhibit alpha-glucosidase and alpha-amylase [42], interrupting the catabolism of dietary starch and decreasing carbohydrate absorption. The decrease in glycemia is decisive for the management of diabetes. Similarly, a “non-hypercaloric” post-prandial state, as a consequence of the reduction in glucose absorption, leads to a decrease in insulin demand, subsequently inhibiting the activity of enzymes involved in the synthesis of fatty acids and of cholesterol, such as acetyl Coenzyme A carboxylase and hydroxy-3-methyl glutaryl Coenzyme A Reductase (HMG-CoA), respectively [43]. Thus, the inhibition of triglyceride and cholesterol anabolism makes quinoa have a protective effect on dyslipidemia. Furthermore, a study reported that a mouse diet supplemented with 3% quinoa pericarp (which contains polysaccharides such as pectins) reduced serum and liver cholesterol [44]. This can be explained by the fact that dietary fiber can bind to bile acid, which increases cholesterol catabolism and fiber fermentation in the colon to produce short-chain fatty acids, which in turn contribute to decreasing cholesterol synthesis in the liver [37].

Furthermore, quinoa protein prevented the increase in plasma and liver cholesterol when mice were fed a cholesterol-supplemented diet. This hypocholesterolemic effect of isolated quinoa protein was correlated with bile acid excretion, probably related to an inhibitory effect of quinoa proteins on bile acid reabsorption in the small intestine, and the control of cholesterol synthesis and catabolism by decreasing HMG-CoA synthesis, while the expression of cholesterol-7 alpha-hydroxylase, a cholesterol catabolic enzyme, was increased [45]. Finally, phytosterols and even saponins in quinoa may also contribute to the decrease in dietary cholesterol because quinoa interferes with intestinal cholesterol absorption and increases fecal bile acid and neutral sterols [46]. On the other hand, the amino acids present in quinoa protein, such as anionic charges, aromatics, cysteine, and phenolic compounds, could be associated with the reduction in lipid peroxidation and LDL oxidation, which are characteristics involved in the development of metabolic and cardiovascular diseases [47].

### 4.3. Bioactive Peptide Generation and Antimicrobial Properties

The liberation of bioactive peptides from quinoa proteins emerges as a critical prerequisite for microbiome modulation, with our analysis revealing two primary pathways: controlled enzymatic hydrolysis and microbial fermentation. As established by a recent comprehensive review, bioactive peptides exhibit beneficial bodily functions and contribute to a healthy gastrointestinal system by influencing barrier functions, immune responses, and gut microbiota [48]. Notably, the preference for LAB fermentation in the literature reflects both its efficiency in peptide release and the concurrent generation of valuable postbiotic metabolites.

Furthermore, the antimicrobial properties of quinoa-derived peptides represent a crucial mechanism for microbiome modulation. Studies demonstrate effectiveness against pathogenic bacteria, including *Escherichia coli* and *Staphylococcus aureus* [12,32], which suggests that these peptides contribute to microbiome health through selective antimicrobial activity. According to Mazurkiewicz et al. (2023) [49], bioactive antimicrobial peptides (BAPs) can kill pathogenic microorganisms by disrupting membrane integrity, inhibiting DNA and RNA synthesis, preventing protein synthesis, blocking protein activity, or interacting with certain intracellular targets [50]. In addition, the positive effects of BAP consumption extend to gut microbiota modulation and affect the equilibrium of reactive oxygen species in the gut. However, the reviewed studies did not typically report on the strain-specificity of these antimicrobial effects, nor did they assess potential off-target impacts on beneficial commensal bacteria. Investigating this is a crucial next step for ensuring the safe application of these peptides.

Remarkably, the methodological evolution toward in silico approaches and enzymatic hydrolysis represents a significant advancement in peptide generation efficiency. These newer techniques achieve higher innovation scores while requiring reduced experimental durations [35], thereby suggesting a transition toward more sustainable and scalable production methods. Nevertheless, traditional fermentation approaches remain valuable for applications within food matrices, where the generation of complex peptide profiles and postbiotic compounds provides additional functional benefits [22]. It is important to note that this review found no studies that conducted a direct head-to-head comparison of these different peptide generation methods (e.g., fermentation vs. enzymatic hydrolysis) on microbiome modulation outcomes. Such comparative studies are a critical knowledge gap and are needed to optimize peptide production for specific functional effects.

### 4.4. SCFA Production and Metabolic Implications

The consistent enhancement of SCFA production, particularly butyrate, represents one of the most significant findings of this review. As Huang et al. (2024) [5] explained, quinoa protein, when digested in the hindgut, yields undigested proteins and amino acids that can be utilized to produce SCFAs in the colon, thereby lowering intestinal pH and regulating microbiota growth in vivo. Consequently, this metabolic transformation is crucial for maintaining intestinal homeostasis and supporting beneficial microbial communities.

Quantitatively, Zeyneb et al. (2021) [27] demonstrated that after 24 h of anaerobic incubation, the total SCFAs of cooked quinoa, uncooked quinoa, and quinoa polysaccharides were 82.99, 77.11, and 82.73 mM, respectively, thus showing substantial metabolite production across different quinoa preparations. The preferential enhancement of butyrate over other SCFAs suggests metabolic bias toward fermentative pathways that are particularly beneficial for gut health, including maintenance of epithelial barrier function and modulation of immune responses [11]. Notably, some of the included studies established a direct correlation between these SCFA increases and host physiological benefits. For instance, Liu et al. (2022) [24] demonstrated that the butyrate-promoting effects of quinoa bran fiber were directly linked to improvements in intestinal barrier integrity markers in a colitis model, providing a mechanistic link between microbial metabolism and host health.

Moreover, the physiological implications of enhanced SCFA production extend beyond local gut effects. According to recent evidence, these degradation products can alter microbial diversity and promote the production of beneficial metabolites, contributing to overall health [51]. SCFAs serve as signaling molecules in the gut–brain axis, influence systemic metabolism through effects on gluconeogenesis and lipogenesis, and modulate immune function through interactions with G-protein coupled receptors [52,53].

### 4.5. Comparative Analysis with Other Plant-Derived Peptides

The gut microbiome modulatory effects of quinoa-derived peptides demonstrate unique characteristics when compared to other plant protein sources. While soy peptides have been extensively studied for their isoflavone content and estrogenic effects, Wijesekara, 2024 [48] noted that peptides and proteins from several animal and plant sources have been widely explored in relation to gut microbiome modulation; however, the effects of soy peptides and other soy derivatives on gut microbiota remain largely unexplored. In contrast, quinoa’s advantage lies in its complete amino acid profile and absence of anti-nutritional factors that may limit the bioavailability of bioactive compounds [54].

Additionally, emerging research suggests that peptides can modulate the intestinal microenvironment by regulating specific gut microbiota species, such as lactic acid bacteria, bifidobacteria, and yeasts [38]. The selectivity of quinoa peptides for beneficial microbial taxa, combined with their antimicrobial effects against pathogens, positions them as particularly valuable functional ingredients for microbiome-targeted interventions compared to other plant-derived alternatives.

### 4.6. Clinical Translation Challenges and Opportunities

Despite promising preclinical evidence, several challenges must be addressed to enable clinical translation of quinoa-derived bioactive peptides. The heterogeneity in study designs, intervention preparations, and outcome measures complicates direct comparisons and limits the ability to establish standardized protocols for human applications. Thus, while it is evident that quinoa has multiple properties for controlling biomarkers associated with disease risks, it is not possible to affirm that quinoa consumption is effective against a specific disease. As Wang et al. (2022) [25] emphasized, while dietary bioactive peptides positively impact gastrointestinal homeostasis by modulating barrier function, immune responses, and gut microbiota, there remains limited clinical evidence on the safety and efficacy of bioactive peptides, much less on the applications of dietary peptides for the treatment or prevention of diseases related to the gastrointestinal tract.

Consequently, the optimization of peptide generation methods emerges as a critical consideration for commercial applications. While in vitro fermentation approaches provide valuable mechanistic insights, scalable production methods must balance efficiency, cost-effectiveness, and retention of bioactivity. The evolution toward enzymatic hydrolysis and in silico prediction methods suggests promising directions for industrial-scale production of standardized quinoa peptide preparations [34].

Furthermore, bioavailability and stability represent additional challenges requiring targeted investigation. Quinoa peptides must survive gastric digestion and maintain biological activity in the complex intestinal environment to exert their modulatory effects. As Parvez et al. (2024) [50] suggested, it is possible to consider controlled delivery systems such as microparticulate, nanoemulsion, and nanostructured lipid carriers or chemical modifications such as the cyclization of bioactive peptide structures that are vulnerable to digestive enzymes or thermal treatment.

### 4.7. Implications for Functional Food Development

The evidence synthesized in this review supports the potential for quinoa-based functional food development targeting gut health applications. The combination of nutritional completeness, bioactive peptide content, and demonstrated microbiome modulatory effects positions quinoa as an ideal substrate for functional ingredient development. As previously established, in vitro studies have suggested that quinoa has a prebiotic effect, including promoting the growth of beneficial bacteria and the production of SCFAs [39].

Moreover, the disease-specific modulation patterns observed across different experimental models suggest opportunities for targeted therapeutic applications. Products designed for metabolic health could leverage quinoa’s capacity to promote lean microbial profiles and enhance SCFA production, while anti-inflammatory formulations could target the reduction in pathogenic taxa and enhancement of barrier function [23,25].

Nevertheless, standardization of quinoa peptide preparations emerges as a crucial requirement for functional food applications. The variability in bioactive peptide content across quinoa varieties, processing methods, and extraction techniques necessitates the development of standardized protocols ensuring consistent therapeutic efficacy. This includes optimization of fermentation conditions, hydrolysis parameters, and quality control measures for bioactive peptide quantification [20,21].

### 4.8. Future Perspectives and Research Directions

Several critical research gaps must be addressed to advance the field toward clinical applications. Human clinical trials represent the most pressing need, with carefully designed studies required to validate the microbiome modulatory effects observed in preclinical models. These studies should incorporate standardized quinoa peptide preparations, validated microbiome analysis methods, and clinically relevant endpoints, including functional digestive health measures and systemic biomarkers of metabolic function. Specifically, target populations could include individuals with metabolic syndrome, non-alcoholic fatty liver disease (NAFLD), or mild-to-moderate inflammatory bowel disease (IBD) in remission. Key clinical endpoints should extend beyond microbiome metrics to include validated clinical markers such as HbA1c, HOMA-IR, serum lipids, C-reactive protein (CRP), and fecal calprotectin, thereby linking microbiome modulation to tangible health outcomes.

Concurrently, mechanistic studies are needed to elucidate the specific molecular pathways through which quinoa peptides modulate microbial communities. As Wijesekara (2024) [48] noted, the interplays between bioactive peptides and gut microbiota are not fully understood; however, bioactive peptides hold promise as modulators of the gut microbiota to promote gut health. Advanced approaches, including metabolomics, transcriptomics, and proteomics, could provide insights into the cellular and molecular mechanisms underlying the observed effects. To understand the potential systemic effects of these peptides, future research should include pharmacokinetic and pharmacodynamic (PK/PD) studies. Assessing the in vivo absorption, distribution, metabolism, and excretion (ADME) of quinoa peptides is essential to determine if they reach systemic circulation and exert bioactivity beyond the gastrointestinal tract.

Furthermore, the development of personalized nutrition approaches represents an emerging opportunity in this field. Individual variations in baseline microbiome composition, genetic factors affecting protein metabolism, and environmental influences may modulate responses to quinoa peptide interventions. Understanding these factors could enable the development of personalized recommendations optimizing therapeutic outcomes for specific population groups or disease states [55,56].

### 4.9. Study Limitations

Several limitations of this scoping review and the underlying evidence base must be acknowledged.

First, the significant methodological heterogeneity across studies, spanning experimental models, intervention preparations, and analytical methods (including different metrics for alpha diversity and different quantification techniques for SCFAs), precluded any quantitative meta-analysis and limited the ability to establish clear dose–response relationships. This is a critical point, as interventions ranged from whole quinoa grain to isolated fibers and protein hydrolysates, making it difficult to attribute the observed effects solely to bioactive peptides versus the synergistic action of multiple quinoa components like fibers and polyphenols. Disentangling these effects remains a key challenge for future research.

Second, the generalizability of the findings is constrained by a geographical clustering of research, with 42% of studies originating from China, and the predominance of specific animal models (e.g., DSS-induced colitis, high-fat diet-induced obesity). Factors such as quinoa cultivars, local dietary habits, and baseline microbiome compositions can vary significantly by region, potentially influencing outcomes and limiting the translational scope to other conditions.

Furthermore, as stated in our methods, a formal risk-of-bias assessment was not performed, which is consistent with the scoping review framework. However, a qualitative appraisal of the primary research suggests that many studies did not fully report on randomization, blinding, or sample size calculations, which introduces a potential risk of bias in the evidence base.

Finally, the variable quality of microbiome analysis methods across the included articles, combined with the exclusive focus on preclinical evidence, underscores the exploratory nature of the current literature. Consequently, the absence of human clinical trials represents the most significant knowledge gap that must be addressed before these findings can be translated to human applications. This review did not systematically extract data on the specific sequencing platforms (e.g., 16S rRNA gene variable region) or bioinformatics pipelines used, and this variability could influence taxonomic resolution and the direct comparability of findings at the genus level.

## 5. Conclusions

This scoping review provides compelling preclinical evidence for the capacity of quinoa-derived bioactive peptides and proteins to modulate the gut microbiome in beneficial ways. The consistent patterns of enhanced microbial diversity, promotion of beneficial taxa, increased SCFA production, and disease-specific modulatory effects across diverse preclinical models support the potential for quinoa as a functional ingredient targeting gut health applications. The mechanistic insights revealed through this synthesis provide a foundation for the rational design of quinoa-based therapeutic interventions. However, it is crucial to emphasize that these findings are preclinical. Clinical translation requires the standardization of production methods, validation through human trials, and the development of appropriate delivery systems, ensuring bioavailability and stability.

The growing understanding of gut microbiome contributions to human health, combined with the demonstrated modulatory capacity of quinoa-derived peptides, positions this field for significant advancement. Therefore, future research should prioritize human clinical validation, mechanistic elucidation, and the development of standardized therapeutic preparations to realize the full potential of quinoa as a functional food ingredient for gut health promotion.

## Figures and Tables

**Table 1:** General characteristics of the included primary studies.

Reference (Author, Year, Country)	Main Objective	Experimental Model	Main Intervention
Ayyash et al. (2018), UAE [21]	To investigate bioactivity generation through quinoa fermentation.	In vitro (Solid-state fermentation).	Quinoa flour fermented with *Bifidobacterium*.
Bianchi et al. (2014), Brazil [31]	To evaluate the effect of a quinoa beverage in a human GI ecosystem simulator.	In vitro (SHIME^®^ simulator).	Fermented soy–quinoa (30%) beverage.
Cizeikiene et al. (2021), Lithuania [32]	To evaluate the effect of lactic fermentation on quinoa characteristics.	In vitro (LAB fermentation).	Quinoa flour fermented with *Lactobacillus*.
Fan et al. (2023), China [33]	To study the preparation and stability of antihypertensive quinoa peptides.	In vitro (Fermentation).	Quinoa flour fermented with *L. paracasei*.
Gullón et al. (2016), Spain/Portugal [26]	To evaluate the prebiotic effect of quinoa.	In vitro (Human fecal fermentation).	Cooked quinoa flour (post-digestion).
Mahdavi-Yekta et al. (2023), Iran [34]	To evaluate the antimicrobial activity of quinoa protein hydrolysate.	In vitro (Agar diffusion assay).	Quinoa protein hydrolysate (QHP).
Menfaatli et al. (2024), Turkey [35]	To evaluate in silico the antimicrobial potential of quinoa peptides.	In silico (Simulated digestion).	Predicted quinoa peptides.
Rizzello et al. (2016), Italy [36]	To evaluate the use of quinoa sourdough to improve bread.	In vitro (Fermentation).	Quinoa flour sourdough.
Rizzello et al. (2017), Italy [22]	To enhance quinoa antioxidant properties through fermentation.	In vitro (Fermentation).	Quinoa flour fermented with *L. plantarum*.
Vilcacundo et al. (2018), Ecuador [20]	To evaluate the properties of quinoa peptides.	In vitro (Simulated GI digestion).	Digested quinoa protein.
Zeyneb et al. (2021), China [27]	To study the effect of quinoa on human microbiota.	In vitro (Human fecal fermentation).	Raw/cooked quinoa (post-digestion).

**Table 2:** General experimental conditions of studies with mice fed quinoa.

Reference (Author, Year, Country)	Main Objective	Experimental Model	Main Intervention	Dose (% of Diet, *w*/*w*)	Time of Administration
Fotschki et al. (2020), Poland [11]	To evaluate the effect of quinoa flours on intestinal microbial activity.	In vivo (Wistar rats).	Quinoa protein-rich flour.	28% daily	2 weeks
Guo et al. (2021), China [38]	To evaluate the effect of quinoa protein on blood pressure and microbiota.	In vivo (SHR hypertensive rats).	Quinoa protein (QP).	100 mg/kg, 200 mg/kg, and 400 mg/kg	2 weeks
Li et al. (2025), China [23]	To evaluate the effect of quinoa on diabetes and microbiota.	In vivo (db/db diabetic mice).	Quinoa.	60% and 100%	9 weeks
Liu et al. (2018), China [39]	To investigate the effect of quinoa on colitis and intestinal dysbiosis.	In vivo (DSS-induced colitis mice).	Whole quinoa grain.	907 g/kg	10 days
Liu et al. (2022), China [24]	To study the effect of quinoa fiber on colitis and microbiota.	In vivo (DSS-induced colitis mice).	Quinoa bran soluble dietary fiber (QBSDF).	1.5 g/kg	2 weeks
Noratto et al. (2019), USA [37]	To investigate the effect of quinoa on cholesterol metabolism.	In vivo (db/db diabetic mice).	Whole quinoa grain.	125 g/kg	8 weeks
Wang et al. (2022), China [25]	To investigate the mechanisms of quinoa in obesity.	In vivo (HFD mice).	Whole quinoa grain.	2 g/day	6 weeks
Fan et al. (2023), China [33]	To study the effect of quinoa on colorectal cancer.	In vivo (C57BL/6 mice)	Digested quinoa protein.	100 and 400 mg/kg/day	5 days
An et al. (2021), China [40]	To evaluate the effect of quinoa on metabolism and dysbiosis in obese mice.	In vivo (HFD mice).	Whole quinoa grain (with/without saponins).	2 g/day	12 weeks

Abbreviations: DSS, Dextran Sulfate Sodium; HFD, High-Fat Diet; QP, Quinoa Protein; QBSDF, Quinoa Bran Soluble Dietary Fiber; SHR, Spontaneously Hypertensive Rats; USA, United States of America.

**Table 3:** Summary of effects on microbiota composition in in vivo studies.

Reference (Author, Year)	Animal Model	Intervention	Effect on Alpha Diversity	Key Modulated Taxa
An et al. (2021) [40]	HFD mice	Whole grain	Not reported	↑ *Bacteroidetes*, ↑ *Actinobacteria*; ↓ F/B ratio.
Guo et al. (2021) [38]	Hypertensive rats	Quinoa protein	Increased	↑ *Turicibacter*, ↑ *Allobaculum*.
Li et al. (2025) [23]	Diabetic mice	Whole grain	Increased	↑ *Lactobacillus*, ↑ *Faecalibaculum*; ↓ *Helicobacter*.
Liu et al. (2018) [39]	Colitis mice	Whole grain	Increased	↓ *Proteobacteria*, ↓ *Escherichia/Shigella*.
Liu et al. (2022) [24]	Colitis mice	Bran fiber	Increased	↑ *Lachnospiraceae* (butyrate producer).
Wang et al. (2022) [25]	HFD mice	Whole grain	Not reported	↑ *Blautia*; ↓ F/B ratio.

Abbreviations: HFD, high-fat diet; F/B ratio, Firmicutes/Bacteroidetes ratio. The symbols ↑ and ↓ indicate an increase and a decrease, respectively.

**Table 4:** Summary of effects on metabolic and functional activity.

Reference (Author, Year)	Experimental Model	Effect on SCFAs	Other Functional Results
Bianchi et al. (2014) [31]	In vitro (SHIME^®^)	↑ Butyrate, ↑ Propionate	↓ Ammonia.
Fotschki et al. (2020) [11]	In vivo (Rats)	↑ Total SCFAs, ↑ Butyrate	↓ Cecal pH; ↑ Microbial enzymatic activity.
Gullón et al. (2016) [26]	In vitro (Fecal)	↑ Acetate	Not measured.
Liu et al. (2022) [24]	In vivo (Mice)	↑ Butyrate	↑ Intestinal barrier integrity.
Zeyneb et al. (2021) [27]	In vitro (Fecal)	↑ Total SCFAs	↓ pH.

↑—increase; ↓—decrease.

**Table 5:** Summary of studies on the biological activity of peptides obtained from the enzymatic hydrolysis of quinoa protein.

Reference (Author, Year)	Peptide Production Method	Demonstrated Bioactivity	Target Bacteria	Duration (Hours)
Ayyash et al. (2018) [21]	Fermentation with *Bifidobacterium*.	Release of peptides with antihypertensive activity.	Not applicable.	48
Cizeikiene et al. (2021) [32]	Fermentation with LAB.	Antimicrobial activity.	*E. coli*, *S. aureus*.	72
Fan et al. (2023) [33]	Fermentation with *L. paracasei*.	Antimicrobial activity, digestion stability.	*E. coli*, *S. aureus*.	40
Mahdavi-Yekta et al. (2023) [34]	Enzymatic hydrolysis (pepsin, alcalase).	Antimicrobial activity.	*E. coli*.	2.5–3.5
Menfaatli et al. (2024) [35]	In silico digestion (pepsin).	Predicted antimicrobial and antibiofilm activity.	*H. pylori*.	Not applicable.
Rizzello et al. (2016) [36]	Fermentation with LAB (sourdough).	Increased proteolysis.	Not applicable.	16
Rizzello et al. (2017) [22]	Fermentation with LAB.	Release of peptides with antioxidant activity.	Not applicable.	24
Vilcacundo et al. (2018) [20]	Simulated GI digestion (pepsin–pancreatin).	Identification of released peptides (<3 kDa).	Not applicable.	4

Abbreviations: *E. coli*, *Escherichia coli*; GI, Gastrointestinal; *H. pylori*, *Helicobacter pylori*; LAB, Lactic Acid Bacteria; *S. aureus*, *Staphylococcus aureus*.

## Data Availability

The original contributions presented in this study are included in the article. Further inquiries can be directed to the corresponding authors.

## Abbreviations

The following abbreviations are used in this manuscript:

AOM	Azoxymethane
BAPs	Bioactive Antimicrobial Peptides
DSS	Dextran Sulfate Sodium
F/B ratio	Firmicutes/Bacteroidetes Ratio
GI	Gastrointestinal
HFD	High-Fat Diet
JBI	Joanna Briggs Institute
LAB	Lactic Acid Bacteria
PCC	Population, Concept, and Context
PRISMA-ScR	Preferred Reporting Items for Systematic Reviews and Meta-Analyses extension for Scoping Reviews
QBSDF	Quinoa Bran Soluble Dietary Fiber
QHP	Quinoa Protein Hydrolysate
QP	Quinoa Protein
SCFAs	Short-Chain Fatty Acids
SHR	Spontaneously Hypertensive Rats
SHIME^®^	Simulator of the Human Intestinal Microbial Ecosystem
UAE	United Arab Emirates
USA	United States of America
